# Changes in the Essential Oil Composition in the Needles of Scots Pine (*Pinus sylvestris* L.) Under Anthropogenic Stress

**DOI:** 10.1100/tsw.2007.36

**Published:** 2007-03-21

**Authors:** Asta Judzentiene, Aida Stikliene, Eugenija Kupcinskiene

**Affiliations:** ^1^ Institute of Chemistry, A. Gostauto 9, LT – 01108, Vilnius, Lithuania; ^2^ Lithuanian University of Agriculture, Department of Ecology, Studentu 11, LT-53361, Kaunas, Akademija, Lithuania; ^3^ Kaunas University of Medicine, Mickeviciaus 9, LT-44037, Kaunas, Lithuania

**Keywords:** phytoindication, conifers, secondary metabolites, monoterpenes, sesquiterpenes, diterpenes, industrial pollution, cement dust, ammonia pollution

## Abstract

Unfavorable anthropogenic factors, such as air pollution, lead to biochemical responses in trees. Changes in the amounts of secondary metabolites may be early indicators of invisible injuries. The aim of this study was to evaluate composition of the essential oils in the needles of Scots pine (*Pinus sylvestris* L.) growing in the areas affected by pollutant emissions of main factories in Lithuania: a nitrogen fertilizer factory (NFF), a cement factory (CF), and an oil refinery (OR). Totally, 14 pine stands were examined along transects from the factories (July 2005). Volatile components of the needles were extracted and analyzed by GC and GC/MS. Over 70 components of the essential oils were identified in current-year and 1-year-old needles.

Along the CF transect for current-year needles, the percentage of diterpenes was decreasing with the increasing pH of the pine bark (r = -0.582; *p* < 0.05) or with the increasing concentration of SO_2_ (r = -0.573; *p* < 0.05); for 1-year-old needles, the percentage of diterpenes was decreasing with the increasing pH of the bark (r = -0.534; p < 0.05). Along the OR transect, in both the current-year and 1-year-old needles, the percentage of diterpenes was decreasing with the increasing SO_2_ (respectively, r = -0.773; *p* < 0.01; r = -0.486; *p* < 0.05); an opposite relation was true for sesquiterpenes (respectively, r = -0.751; *p* < 0.01; r = 0.785; *p* < 0.01). The view was different along the NFF transect. For current-year needles, the percentage of monoterpenes was decreasing with the increasing NH^3^ (r = -0.669; *p* < 0.01); while the percentage of sesquiterpenes or oxysesquiterpenes was increasing with the increasing NH^3^ (respectively, r = 0.540; *p* < 0.05 and r = 0.688; *p* < 0.01). For each transect, cluster analysis of the percentages of components of essential oils in the needles allowed us to distinguish the most contrasting stands according to the concentration of air pollutants. Current-year needles were more effective as indicators of the effects of pollution than 1-year-old needles in the case of the NFF and the OR transects, and both-aged needles were equally valuable in the case of the CF transect. The changes detected in the proportions of components of the essential oils in the needles of the trees affected by the industrial emissions may play a significant role in modifying the susceptibility of the pine stands to the biotic factors, and also may alter emissions of terpenes from the stands to the atmosphere.

### INTRODUCTION

In many countries in Europe and Northern America, atmospheric pollution decreased in the 1980s and 1990s. The wide scope of ongoing forest monitoring in the areas affected by industrial pollution in Western countries is decreasing through the current decade[1,2].

In Lithuania, since the 1990s, the reduced level of emissions in a cement factory (operating since 1952), a nitrogen fertilizer factory (since 1965), and an oil refinery (since 1980) caused further adverse changes in the surrounding forests[3,4] and some disturbances were registered in the ongoing decade[5].

Unfavorable anthropogenic factors, such as air pollution, lead to biochemical responses in trees. Recently, greater attention started to be paid to the composition of essential oils in conifers, including pine[6]. Wider surveys of the needle essential oil composition under pollutant effects and other stressors are still scarce[7,8,9].

The present study was aimed to evaluate whether lower-level industrial pollution caused by the oil refinery, the cement factory, and the nitrogen fertilizer factory affects the composition of the essential oils in the needles of Scots pine (*Pinus sylvestris* L.) growing under different levels of pollution/at different distances from the pollution source.

### MATERIAL AND METHODS

#### Study Area

The Scots pine *(Pinus sylvestris* L.) growing along the transects from the cement factory (CF), the oil refinery (OR), and the nitrogen fertilizer factory (NFF) in Lithuania were investigated. In 2004–2005, total emissions from the CF comprised up to 3000 t year$^{-1}$, emmisions from the OR 24,000 t year$^{-1}$, emissions from the NFF up to 4000 t year$^{-1}$. The total deposition of calcium and magnesium ions along the CF transect ranged between 19.1 and 2.6 kg ha$^{-1}$ month$^{-1}$ and the pH of the bark of the pines along the transect belonged to interval 6.4–5.0. Sites near the OR differed in the amounts of sulfur dioxide up to 2.4 times (with the highest mean monthly concentration of 27 μg m$^{-3}$). The sites along the transect next to the NFF varied up to 7.4 times in the amounts of aerial ammonia (with the highest mean monthly concentration of 26 μg m$^{-3}$). At the most contrasting (according to the distance) sites of transects, the concentration of $NO_2$ (with the highest mean monthly concentration of 8.2 μg m$^{-3}$) varied by 1.5–2.3 times[2]. In all three transects, the most polluted sites were the ones closest to the factories.

Middle-aged pines on histosols (near the CF), luvisols (near the OR), and arenosols (near the NFF) were examined. Selection of sites was based on availability of the stands and the prevailing wind direction from each pollution source. Related to the CF, four stands belonging to *Carico-sphagno-Pinetum* (siccata) type in a 10-km transect (northeast direction) were studied. Near the OR, four stands of *Oxalido-Pinetum* type in a 5.4-km northeast transect and one stand (3.6 km away from the OR, east direction) were examined. Next to the NFF, four stands of *Vaccinio-myrtillo-Pinetum* type in northeast direction (25-km interval) were chosen and one stand in the opposite direction (15 km away from NFF) was examined.

### Plant Material

Branches were cut from eight pines at the height of 6–8 m above the ground. From each tree, four shoots with the current-year (c) and 1-year-old (c+1) needles were cut in July 2005. For analysis, four independent samples were prepared from each stand. A separate sample was made by mixing equal amounts of the needles collected from eight trees[10] and drying the material at room temperature (20–25°C).

### Oil Isolation and Analysis

Oil yield was conducted by hydrodistillation of 50 g of dry needles. Pale yellow oils were obtained in 0.2–0.6 and 0.2–0.3% of the yield, respectively, in the current-year and 1-year-old needles on a dry mass basis. GC analysis was done by HP 5890(II) chromatograph equipped with FID and capillary column HP-FFAP (30 m × 0.25 mm i. d., film thickness 0.25 μm). Analyses by GC/MS were performed using a HP 5890 chromatograph interfaced to an HP 5971 mass spectrometer (ionization voltage 70 eV) and equipped with a capillary column CP-Sil 8 CB (50 m × 0.32 mm i. d., film thickness 0.25 μm). Other details of oil analyses were described earlier[11,12]. Qualitative analysis was based on a comparison of retention times, indexes with mass spectra libraries (Wiley and NBS 54K), and other corresponding data[13].

### Statistical Analysis

To compare the stands along the transects, dispersion, correlation, and cluster analyses were applied using EXCELL, SPSS, and SAS packages. Error bars in the figures indicate an interval of 95% confidence. Agglomerative Hierarchical Clustering was performed to determine whether the observations can be formed into groups suggested by the data. Method was applied using the standardized data. Hierarchical Clustering was done using average linkage (distance between two clusters is the average distance between pairs of observations, one in each cluster).

## RESULTS AND DISCUSSION

Seventy-one identified components made up to 89.1–95.1% of total oil content. The predominant fraction was found to be monoterpenes (19.0–40.0%), with the major constituents being α-pinene (6.1–26.1%) and δ-3-carene (4.9–22.9%; Figs. 1–3). Among oxygenated monoterpenes (0.4–1.6%), bornyl acetate was the most dominant constituent (0.2–5.6%). Sesquiterpenes formed 16.7–32.4% of the oils; amounts of main sesquiterpenes (E)-caryophyllene and δ-cadinene were 2.9–7.9 and 2.7–8.2%, respectively. α-Cadinol (3.9–9.8%) and epi-α-cadinol with epi-α and α-muurolols (4.0–9.1%) were the major compounds in the oxygenated sesquiterpenes fraction (3.1–11.6%). Diterpenes comprised 0.9–13.5% of the oils. Phenols were the smallest group (0.2–2.0%).

### The Case of the Cement Factory

Along the cement factory transect, the most heavily polluted stand (at a distance of 0.5 km from the factory) had significantly the lowest ($p < 0.002$) percentage of diterpenes in the current-year-needles and the highest ($p < 0.035$) percentage of monoterpenes in 1-year-old needles (Fig. 1). In current-year needles, the percentage of diterpenes was increasing with the increasing distance from the cement factory ($r = 0.675$; $p < 0.001$); it was decreasing with the increasing pH of the bark ($r = -0.582$; $p < 0.05$) or with the

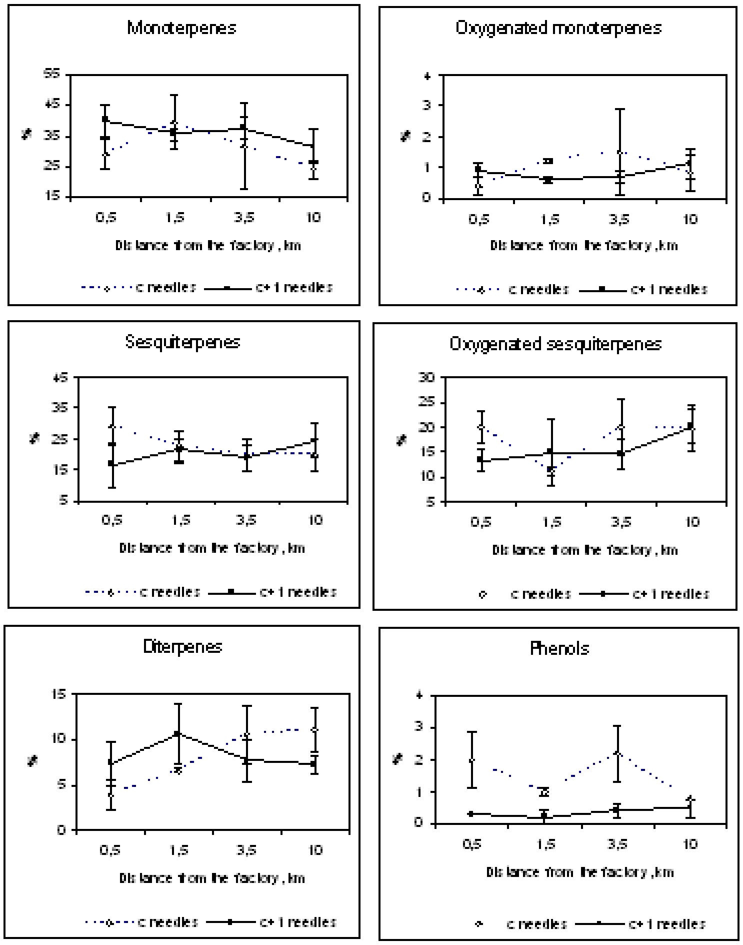

**FIGURE 1.** Percentage composition (mean values, n = 4) of the main classes of essential oils (monoterpenes, oxygenated monoterpenes, sesquiterpenes, oxygenated sesquiterpenes, diterpenes, phenols) in current-year (c) and 1-year-old needles (c+1) of Scots pine (*P. sylvestris* L.) stands growing at different distances from the cement factory (July 10, 2005). Error bars indicate an interval of 95% confidence.

increasing concentration of $SO_2$ (r = –0.573; $p < 0.05$). In 1-year-old needles, the percentage of diterpenes was decreasing with the increasing pH of the bark (r = –0.534; $p < 0.05$); to the contrary, the percentage of monoterpenes was increasing with the increasing pH of the bark (r = 0.625; $p < 0.05$). Grouping of the pine stands along the transect from the cement factory according to the percentage of all components of essential oils in current-year needles showed that the most polluted/the closest to the factory stand was the most distinct (Fig. 4); the same analysis based on 1-year-old needles showed the furthest stand as the most distinct.

### The Case of the Oil Refinery

Along the oil refinery transect, the most heavily polluted stand (a distance of 2.0 km from the factory) had the lowest (by a large degree) percentage of diterpenes in both current-year and 1-year-old needles (respectively, $p < 0.001$; $p < 0.001$) and the highest ($p < 0.017$) percentage of sesquiterpenes in current-year needles (Fig. 2). In current-year needles, the percentage of diterpenes was increasing with the increasing stand distance from the factory (r = 0.522; $p < 0.05$); it was decreasing with increasing $SO_2$ (r =

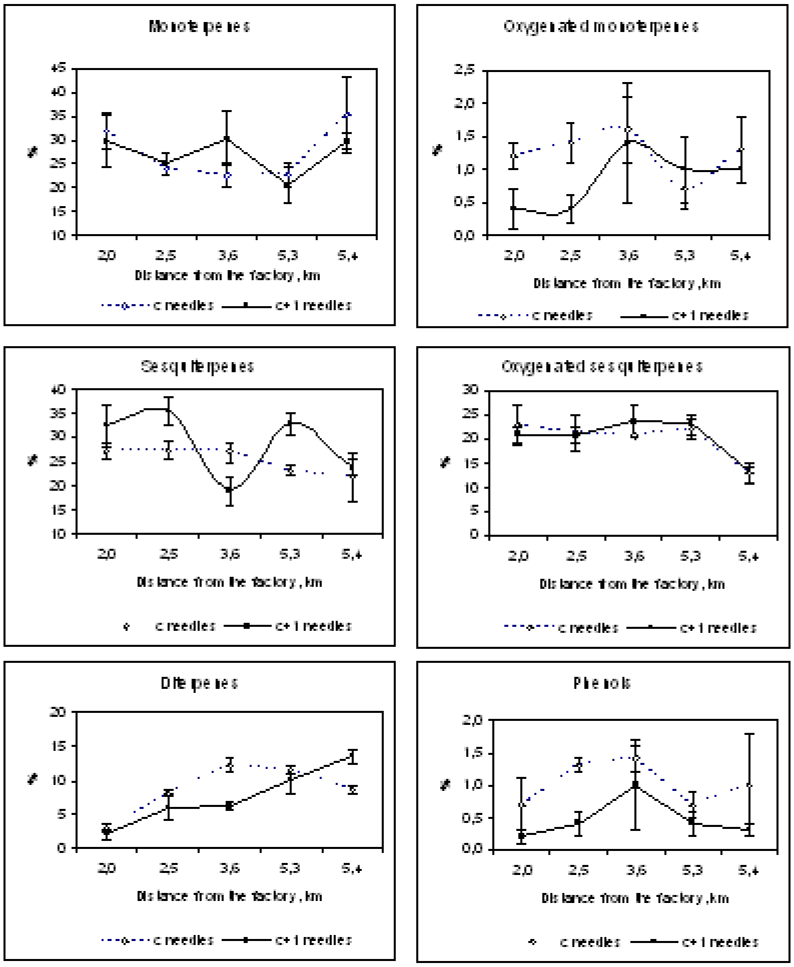

**FIGURE 2.** Percentage composition (mean values, n = 4) of the main classes of essential oils (monoterpenes, oxygenated monoterpenes, sesquiterpenes, oxygenated sesquiterpenes, diterpenes, phenols) in current-year (c) and 1-year-old (c+1) needles of Scots pine (*P. sylvestris* L.) stands growing at different distances from the oil refinery (July 10, 2005). Error bars indicate an interval of 95% confidence.

–0.773; $p < 0.01$). Opposite relations were found between the percentage of sesquiterpenes in current-year needles and the stand distance ($r = –0.586$; $p < 0.05$) or the $SO_2$ concentration ($r = 0.751$; $p < 0.01$). In 1-year-old needles, the percentage of diterpenes was decreasing with increasing $SO_2$ ($r = –0.486$; $p < 0.05$); an opposite relation was found for sesquiterpenes and $SO_2$ ($r = 0.785$; $p < 0.01$). Grouping of the pine stands along the transect from the oil refinery according to the percentage of all components of the essential oils in current-year needles showed that the furthest from the factory stand was the most distinct (Fig. 5); the same analyses based on 1-year-old needles did not show a distribution of stands according to the pollution gradient.

### The Case of the Nitrogen Fertilizer Factory

Along the nitrogen fertilizer factory transect, the most heavily polluted stand (a distance of 2.5 km from the factory) had the lowest (by a significant degree) percentage of monoterpenes ($p < 0.001$), the highest ($p < 0.021$) percentage of oxygenated sesquiterpenes, one of the highest percentages ($p < 0.01$) of sesquiterpenes in current-year needles (Fig. 3). In current-year needles, the percentage of monoterpenes

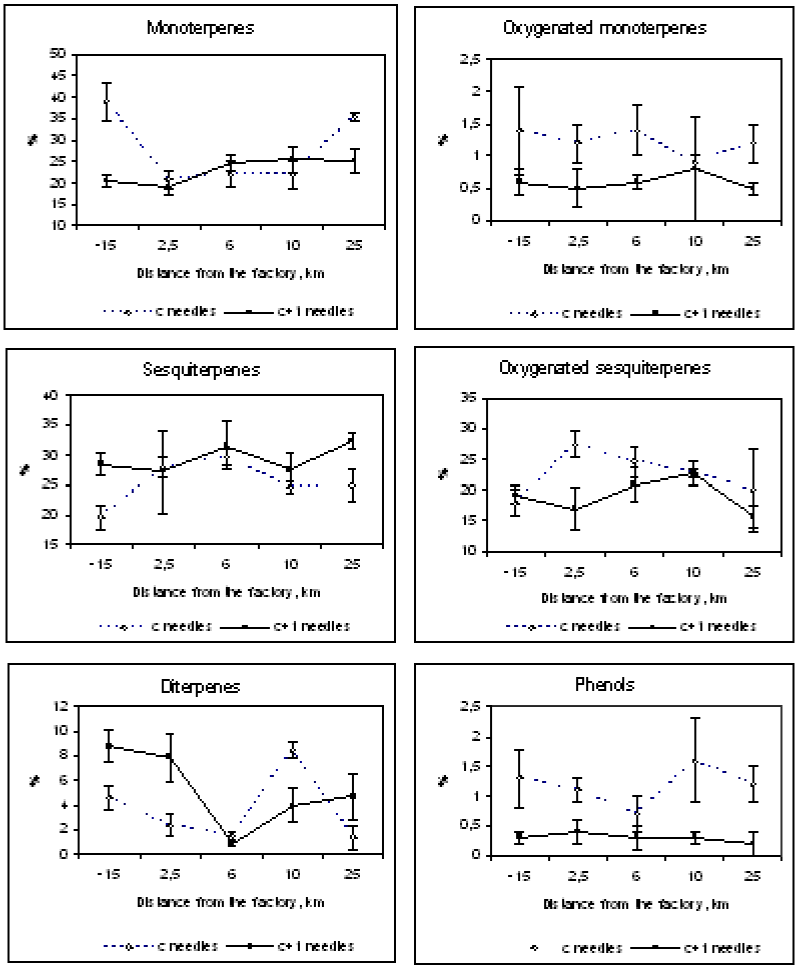

**FIGURE 3.** Percentage composition (mean values, n = 4) of the main classes of essential oils (monoterpenes, oxygenated monoterpenes, sesquiterpenes, oxygenated sesquiterpenes, diterpenes, phenols) in current-year (c) and 1-year-old (c+1) needles of Scots pine (*P. sylvestris* L.) stands growing at different distances from the nitrogen fertilizer factory (July 10, 2005). Error bars indicate an interval of 95% confidence.

was decreasing with the increasing $NH_3$ ($r = -0.669$; $p < 0.01$), while the percentage of sesquiterpenes or oxysesquiterpenes was increasing with the increasing $NH_3$ (respectively, $r = 0.540$; $p < 0.05$ and $r = 0.688$; $p < 0.01$). The grouping of the pine stands along the transect from the nitrogen fertilizer factory according to the percentages of all components of the essential oils in current-year needles showed that the furthest stands (growing at a 25-km distance according to the prevailing wind and at a 15-km distance according to the opposite wind direction) were distinct from the stands located closer to the factory (Fig. 6). The same analysis of 1-year-old needles did not show a distribution of the stands according to the pollution gradient.

Generally, our results concerning the composition of the essential oils in the needles of *P. sylvestris* were similar to those reviewed by Lawrence[14]. Up to now, the knowledge concerning the effects of adverse environmental factors on the proportions between the separate components of the essential oils is contradictory[6,15,16]. There were reported changes in the terpene production of several conifers under the effect of contaminated atmosphere[8,17] suggesting that terpenes might play a role in protection against various industrial (either acidic or alkaline) pollutants. Continuous stress on the pines due to the

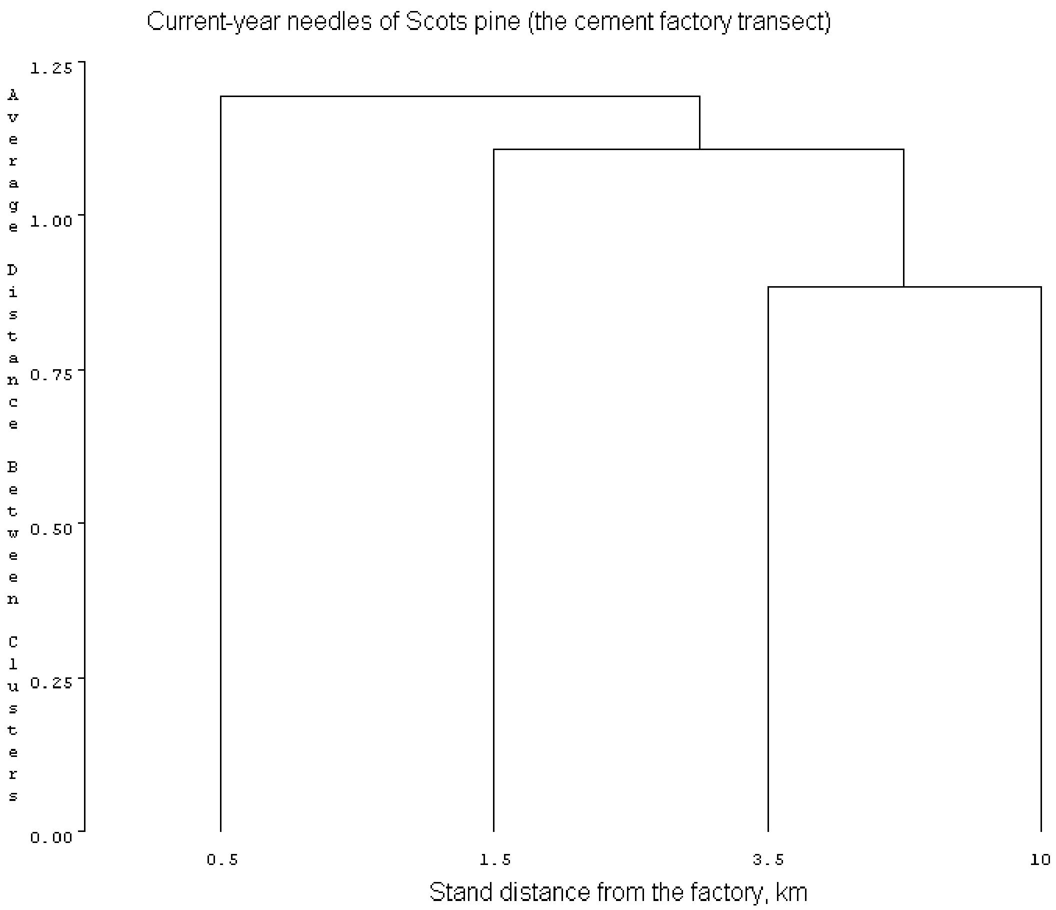

**FIGURE 4**. Grouping of Scots pine (*P. sylvestris* L.) stands growing at different distances from the cement factory according to percentage composition (n = 4) of the components (71) of essential oils in current-year needles (July 10, 2005)

factories may induce, in part, the needle differences observed in the percentage of separate components of the essential oils between stands along transects in Lithuania.

In our assessment, the applied cluster analysis of the percentages of essential oils in the needles showed that the bigger effects of industrial pollution were observed for current-year needles compared to 1-year-old needles in the case of pine stands near the oil refinery and the nitrogen fertilizer factory. For the cement factory transect, both needle age classes demonstrated significant factory distance-related changes, confirming the opinion about the absence of threshold for dust effects[5,18]. Earlier examinations of the same transects showed that worsening of general tree condition and nutritional disturbances could be related to the effects of the factories[2].

Changes in leaf primary and secondary metabolite chemistry have been associated with defoliation in conifers[19,20]. In our study, we observed greater changes in the essential oil composition in the needles for the stands (the nearest to the cement factory and the oil refinery) that had the higher defoliation and the shorter needle retention[2,3,4,5].

Relations between the nutrient availability and the secondary plant compounds in Scots pine have been described in other studies[6,21]. Our results obtained in the former cement factory and the oil refinery transect studies concerning nutrition of Scots pine[4] are consistent with those that have reported higher volatile terpenoid concentrations in the leaves of several plant species growing in conditions with low nitrogen and phosphorus availability[6].

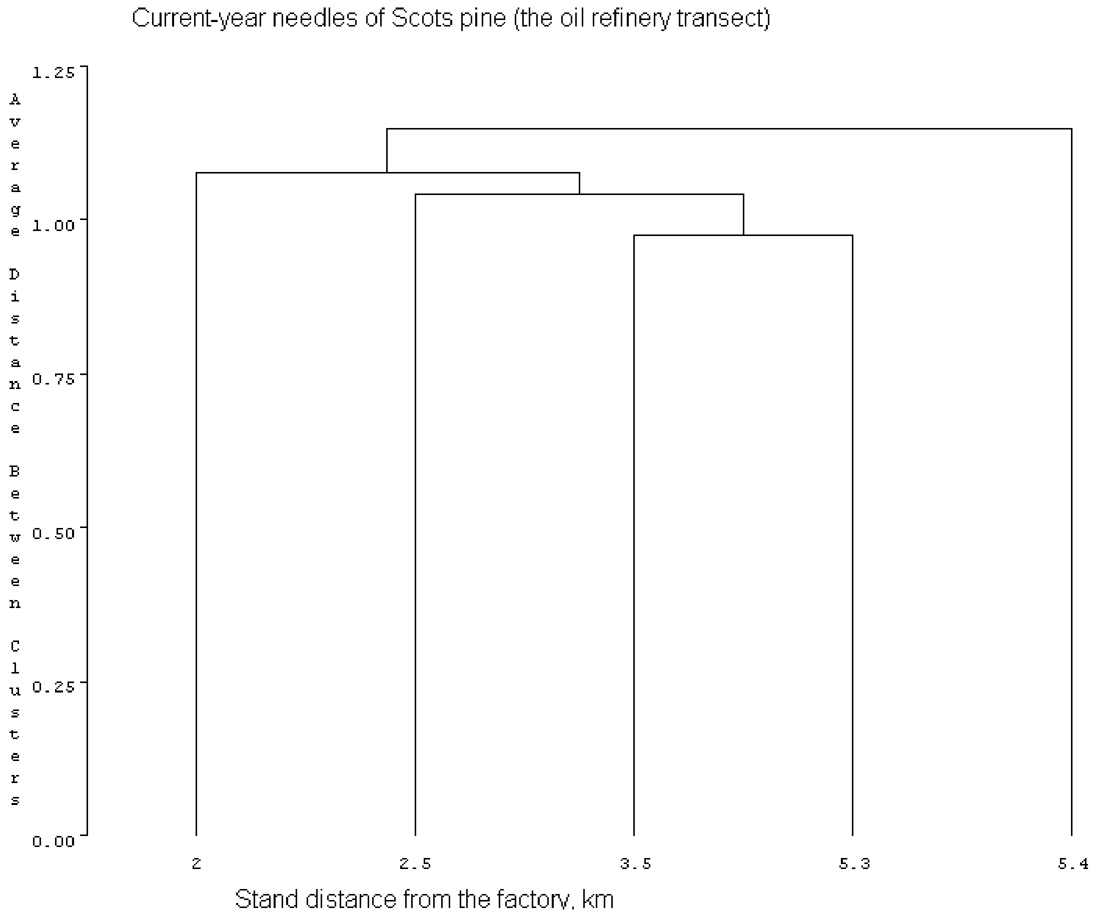

**FIGURE 5**. Grouping of Scots pine (*P. sylvestris* L.) stands growing at different distances from the oil refinery according to percentage composition (n = 4) of the components (71) of essential oils in current-year needles (July 10, 2005)

Because pollen and seeds of the pine are dispersed by wind, the role of the essential oils in the needles cannot be related to the attraction of insects and might be quite unambiguously connected with protection against pathogens and also parasites. The essential oils or their components have been shown to exhibit antiviral, antibacterial, antimycotic, antitoxigenic, antiparasitic, and insecticidal properties, and activity against mite pests and nematodes[6,8]. Changes in the proportions of the components of the essential oils differing in the chain lengths may modify the susceptibility of the polluted (by industrial emissions) pine stands to the biotic factors.

Higher production of the shorter-chain terpenes (observed along the cement factory and oil refinery transects) might be a consequence of tree growth in the polluted environment requiring energy consumption for the reparation processes, while at the same time investing smaller resources for synthesis of protective compounds. The opposite view obtained along the transect from the nitrogen fertilizer factory might be due to the effect of ammonia emitted in low concentrations that are not harmful and even slightly stimulating.

It is recognized that monoterpenes coming from conifer forests forms a significant part in BVOC emissions[22]. Alterations in the proportions of components of essential oils in the needles of pines growing in the areas affected by the pollutants of factories may contribute to the changes of emissions of terpenes.

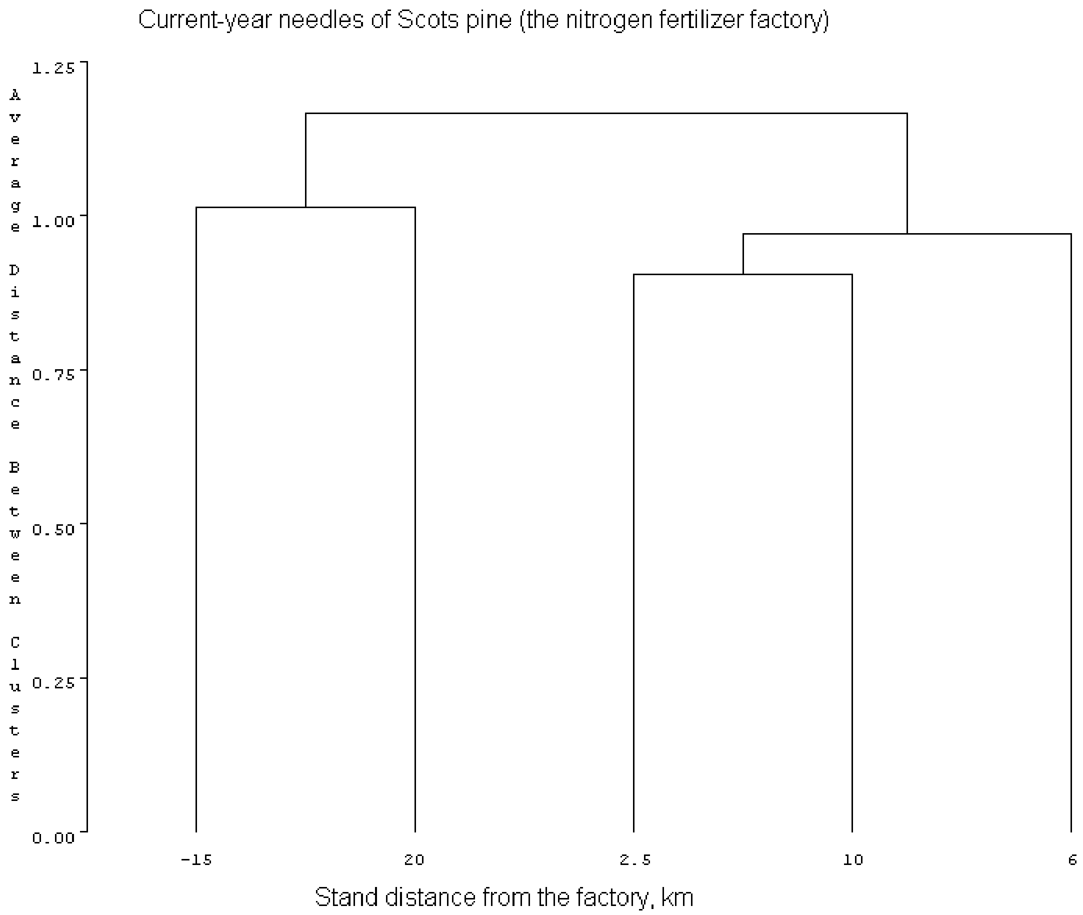

**FIGURE 6**. Grouping of Scots pine (*P. sylvestris* L.) stands growing at different distances from the nitrogen fertilizer factory according to percentage composition (n = 4) of the components (71) of essential oils in current-year needles (July 10, 2005)

### CONCLUSIONS

Under the effect of present lower-level industrial emissions, significant changes occur in the proportions of components of the essential oils in the needles of Scots pine. Correlation analyses show relations between the proportions of classes of the essential oils and the concentrations of air pollutants. According to the cluster analysis for the indication of the effect of pollution on the proportions between components of essential oils, current-year needles were more informative than 1-year-old needles in the case of the cement factory and oil refinery. In general, the pollution from the oil refinery or the cement factory had caused higher proportions of shorter-chain terpenes and lower proportions of longer-chain terpenes; the opposite could be told about the nitrogen fertilizer factory effects.

### ACKNOWLEDGMENTS

This work was supported by the Study and Science Foundation of Lithuania, Project “FIBISTRESS”, the grant No. C-05033/05.
